# Cytokine Profile in Human Peripheral Blood Mononuclear Leukocytes Exposed to Immunoadjuvant and Adjuvant-Free Vaccines Against Influenza

**DOI:** 10.3389/fimmu.2020.01351

**Published:** 2020-06-30

**Authors:** Mikhail Petrovich Kostinov, Nelli Kimovna Akhmatova, Ekaterina Alexandrovna Khromova, Aristitsa Mikhailovna Kostinova

**Affiliations:** ^1^Department of Immunology, I.I. Mechnikov Research Institute of Vaccines and Sera, Moscow, Russia; ^2^Department of Immunology, Sechenov First Moscow State Medical University, Moscow, Russia; ^3^Department of Immunology, NRC Institute of Immunology FMBA of Russia, Moscow, Russia

**Keywords:** vaccines against influenza, subunit immunoadjuvant vaccine, subunit vaccine, split vaccine, cytokine response

## Abstract

**Background:** In the last decade, adjuvant-containing vaccines, exerting different effects on the immune system, including the production of cytokines, which are one of the most important regulatory systems of the body, are introduced into practice.

**Objectives:** An effect of the immunoadjuvant polymer-subunit and adjuvant-free vaccines against influenza on the cytokine profile of mononuclear leukocytes in 27 healthy women was studied.

**Methods:** The study of cytokine profile in human peripheral blood mononuclear leukocytes exposed to vaccines against influenza virus was determined by flow cytometry method (Cytomix FC-500, Beckman Coulter, USA) using the Multiplex-13 test system (Bender MedSystems, Austria).

**Results:** It was established that all the studied vaccines leaded to somewhat increased levels of Th1/Th2/Th17/Th9/Th22 cytokines in the culture fluid of peripheral blood mononuclear leukocytes (PBML), which indicates the activation of both humoral and cellular immunity. An immunoadjuvant vaccine has been shown to be superior in activating the synthesis of Th1 (IL-12, INF-g, IL-2, IL-6, IL-1β, TNF-α) cytokines, IL-9 and IL-22, while the subunit vaccine was superior in activating the synthesis of IL-4, and split vaccine—of IL-5.

**Conclusions:** Immunoadjuvant vaccine is superior in terms of inducing cellular immune effectors to a greater extent compared to subunit and split vaccines.

## Introduction

It is known that in order to successfully eliminate the influenza virus, the immune system uses both innate and acquired effectors that underline their importance not only during natural infection, but also during vaccination (1–6). When the influenza virus is introduced into the body, at the first stage, it is recognized and the innate immune response is triggered in order to prevent virus penetration and multiplying in the epithelial cells of the respiratory tract. Primary recognition of influenza virus in infected cells is performed by pattern-recognizing receptors (1). Signal cascades, started after receptor activation, stimulate the synthesis of pro-inflammatory cytokines and type I interferons (IFN I) (7–9).

IFN I possess strong antiviral activity due to inhibition of protein synthesis in infected cells and limit viral replication. IFN I induce the expression of ISGs genes, which products are able to inhibit viral replication (10–12). Besides, IFN I stimulate dendritic cells (DCs), which enhance presentation of influenza antigens to CD4^+^ and CD8^+^ T-lymphocytes and contribute to the adaptive immune response triggering (13, 14).

While alveolar cells are being infected, alveolar macrophages become activated and phagocyte influenza-infected cells. In addition, activated macrophages begin to produce tumor necrosis factor alpha (TNF-α), which also contributes to the limitation of viral distribution (15, 16). It is known that viremia causes an immune response due to the production of interleukins: IL-1, IL-6, IL-8, TNF-α, immunoglobulins (17, 18).

Cytokines of the interleukin-1 family play an important role in the development of humoral immune response, IL-1β enhances CD40L-mediated activation of human DC, increasing their ability to control IFN-γ secretion in T cells and produce IL-12. L-1β directly affects antigen-specific CD8^+^ T cells, enhancing their expansion, survival, migration, and effector function (19). After inoculation of influenza A virus into rat, murine and human macrophage cell lines, elevated concentrations of IFN I, TNF-α, IL-1, IL-6, and mononuclear cellular chemokine attractants (CCL3/MIP-1α, CCL4/MIP-1β, CCL5/RANTES, CXCL10/IP-10) were detected (20, 21).

The standard of immunogenicity of any vaccines, including influenza, is the immunity that occurs during natural infection (22).

Perhaps the administration of vaccines against influenza can stimulate cytokine production, which should be limited to a moderate degree. It is known that simultaneous increase of TNF-α level with normalization of IL-1β concentration and excessive (hundreds of times) increase of IL-6 in blood serum in patients with pH1N1 influenza virus infection is characterized by aggravation of the condition and the possibility of poor prognosis (23). Changing the profile of Th2-cytokines is important for people with allergopathology, since they have dysregulation of IL-4 secretion: an increase in IgE synthesis in response to IL-4 stimulation leads to an increase in IgE-stimulated cytokine synthesis by mast cells capable of producing IL-4, I1-5, and IL-6 (24).

In the last decade, adjuvant-containing vaccines were introduced into practice. Such products either by providing better recognition of antigens, or by enhancing the immune response, provide a good postvaccinal effect. The use of an immunomodulator as an adjuvant leads to an expansion of the spectrum of immune responses involved in the creation of immune protection (25–30). Immunomodulators differ from each other in origin and mechanism of action. For example, the immunomodulatory effect of adjuvants such as lipopolysaccharide and muramyldipeptide is explained by the involvement of innate immune receptors in this process, followed by stimulation of immune cells (31). However, managing the process of stimulating immune cells using pathogen-associated molecular patterns (PAMP) is very complex. Failure of control can result either in insufficient formation of the immune response, or in the development of an undesirable reaction. In this regard, adjuvants that are not related to PAMP are of greater interest, which include Azoximer bromide, an adjuvant that is widely used in the Russian Federation as part of influenza vaccines. Studies have shown that the use of the Azoximer bromide adjuvant in the vaccine demonstrates a good effect and low reactogenicity, including in women in the second trimester of pregnancy. The use of an anti-influenza vaccine containing Azoximer bromide in pregnant women caused a more pronounced immune effect than when using a subunit adjuvant-free vaccine and did not affect the course of pregnancy and fetal development (26, 27, 32–35).

It can be assumed that the difference in the effectiveness of influenza vaccines may be due to the effect of the adjuvant on the activation of immune effectors, which, as shown above, is closely related, among other things, to the cytokine milieu. Therefore, in order to better understand the mechanism of action of the adjuvant and enhance the ability to control the immune response, the study of the cytokine profile of the adjuvant vaccine is of great interest.

### Study Objective

To investigate the effect of the immunoadjuvant polymer-subunit and adjuvant-free influenza vaccines on the cytokine profile of mononuclear leukocytes.

## Materials and Methods

A single-center, open, non-randomized study of cytokine profile in human peripheral blood mononuclear leukocytes exposed to vaccines against influenza virus.

### Patient Description

Twenty-seven subjects, corresponding to the inclusion criteria, participated in the study.

#### Inclusion Criteria

Eighteen to forty years old women, healthy or with comorbidities in the period of remission. They have never been vaccinated against the influenza and have not experienced influenza or other acute respiratory infections within the last 6 months. It was taken venous blood samples for incubation with influenza vaccines.

#### Exclusion Criteria

History of vaccination against influenza, influenza, or other acute respiratory infections within the last 6 months before the study, administration of other vaccines within 30 days before the study, presence of **s**ignificant diseases in exacerbation stage (nervous, cardiovascular, respiratory (including asthma), gastrointestinal, hepatic, metabolic, and hematological diseases), presence of rheumatic, autoimmune diseases, primary or secondary immunodeficiency, history of immunosuppressive therapy, or treatment with immunostimulants.

### Study Vaccines

Influvac (“Abbott biologicals” B.V., Netherlands)—inactivated subunit influenza vaccine, Vaxigrip (“Sanofi Pasteur,” France)—inactivated split-virion influenza vaccine for influenza prevention. These vaccines contain hemagglutinin of the influenza virus type A subtypes A/H1N1 ℵ A/H3N2 (15 μg each) and hemagglutinin of the influenza virus type B (15 μg). Grippol plus (LLC “NPO Petrovax Pharm,” Russia)—trivalent polymer subunit inactivated influenza vaccine. It contains hemagglutinin of the influenza virus type A subtypes A/H1N1 ℵ A/H3N2 and hemagglutinin of the influenza virus type B (5 μg each), and immunoadjuvant Azoxymer bromide (Polyoxidonium) (500 μg). All the vaccines contained current influenza virus strains for epidemiological seasons 2015–2016 and 2016–2017.

The drug product “Grippol plus” is available only in the composition with an adjuvant. There is no similar subunit vaccine with a similar structure containing 5 μg of antigens in one vaccination dose without an adjuvant.

### Characteristics of Adjuvant Used in the Vaccine

In the Russian Federation, the immunomodulator Azoxymer bromide is successfully used in many disorders separately or in combination with vaccination (36–39). Azoximer bromide is a water—soluble synthetic polymer adjuvant class of heterochain polyamines—copolymer of N-oxide 1,4-ethylene piperazine (N-carboxyethyl)-1,4-ethylene piperazine bromide. Pharmaceutical development and preclinical studies of the vaccine adjuvant were conducted for several years in the early 90's of the 20th century. According to literature data, Azoxymer bromide interacts with three lymphocyte subclasses, predominantly binds to monocytes and neutrophils, and to a lesser extent—with lymphocytes, which leads to increased intracellular H2O2 production. Hydrogen peroxide activates the transcription factor NF-kB, which is involved in the regulation of cytokine synthesis. Increased synthesis of pro-inflammatory cytokines IL-1β, IL-6, TNF-α is observed, that direct the immune response to Th1 cell type and do not cause an increase in IgE levels (40). On a number of model compounds, it was shown that the immunogenicity and protective properties of antigens attached to the Azoximer bromide carrier increase 10-fold with an increase in both the antibody and cell-mediated immune response, the synthesis of all classes of protective antibodies (IgM, IgG, IgA) is enhanced, but the synthesis of allergic IgE antibodies is not stimulated. Thus, some synthetic polyions are strong adjuvants and polyclonal activators of B-and T-lymphocytes (41). Grippol® plus is produced according to international GMP and ISO ISO:9001 standards in clean rooms on the filling line with hurdle technology, which allows excluding the mercury-containing preservative (thiomersal) from the composition of vaccine.

Polymer-subunit influenza vaccine is included in the National immunization schedule of the Russian Federation and has been used for more than 20 years. This vaccine has also been successfully used in a number of other countries: Uzbekistan, Iran, Kyrgyzstan, Belarus.

### Laboratory Methods

Peripheral blood mononuclear leukocytes (PBML), preliminary isolated from whole blood in a Ficoll-Urografin density gradient, were studied. 10^6^ cells in 1 mL of RPMI-1640 with Hepes (PanEco, Russia), containing 0.1 mg/mL gentamicin sulfate (Sigma), glutamine (0.32 mg/mL), and 10% thermo-activated fetal calf serum (PanEco, Russia) were incubated with 10 μL of the appropriate vaccine product for 24 h at 37°C in a humidified atmosphere at 5% CO_2_.

Doses of the drug product were selected as follows: an adult has an average of 50 × 10^9^ white blood cells in 5 L of blood, and one vaccination dose of the influenza vaccine is 0.5 ml. From this calculation, 10 μl of vaccine will be required per 1 ml of cells.

Each patient's cells were incubated separately with each influenza vaccine. The control sample was incubated without the presence of the vaccine.

For the subsequent cytokine analysis using Multiplex-13 test system, the culture supernatants were collected and stored at −80°C.

Cytokine (Th1/Th2/Th17/Th9/Th22) level in PBML supernatants was determined after incubation with vaccines against influenza by flow cytometry method (Cytomix FC-500, Beckman Coulter, USA) using the Multiplex-13 test system (Bender MedSystems, Austria) according to the method described in the manufacturer's instructions. A 3-fold measurement of each sample was made.

### Statistical Analysis

Groups were compared using Mann–Whitney rank sum tests for independent samples. *P* ≤ 0.05 were considered statistically significant (STATISTICA 10 software).

## Results

**Figure 1:**
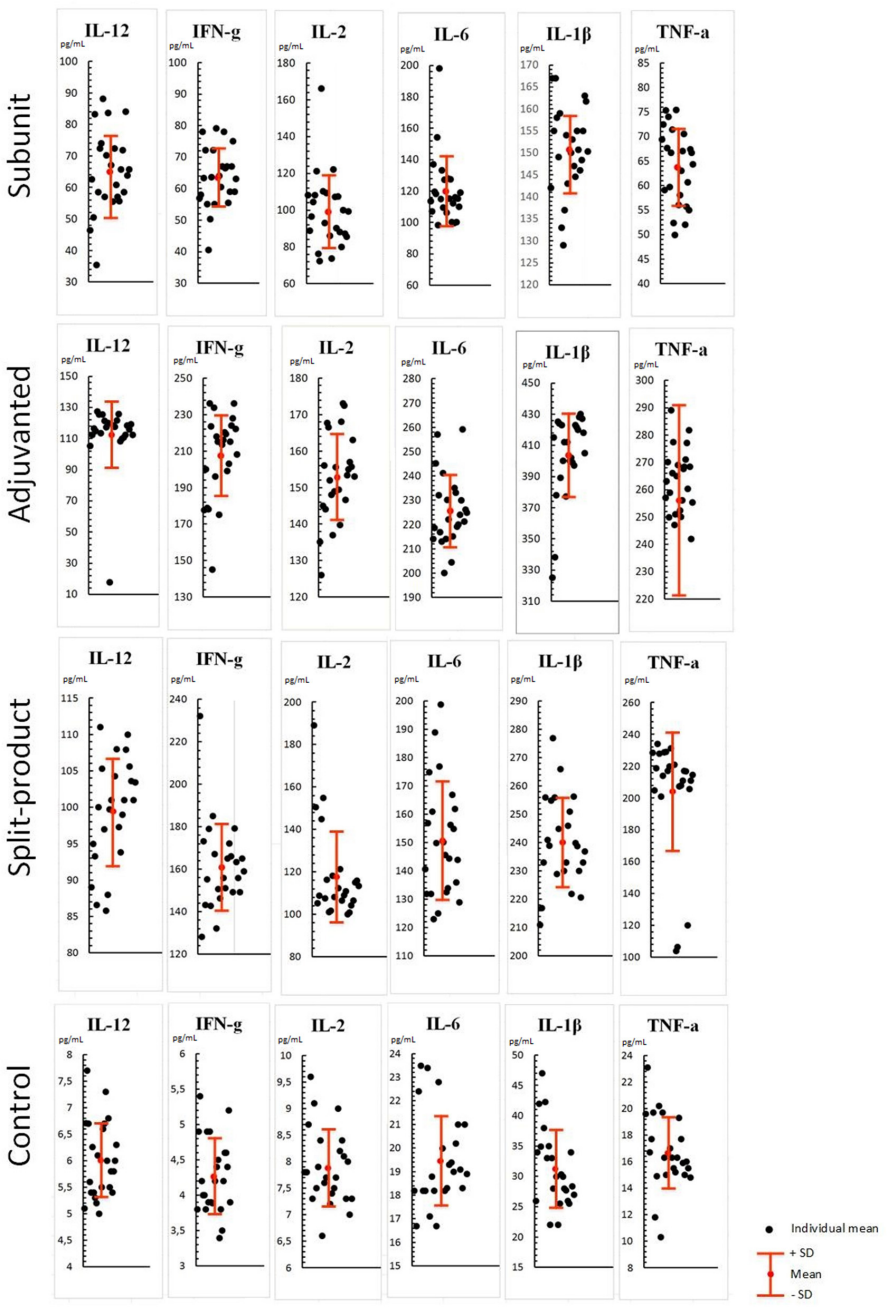
Effect of vaccines against influenza on Th-1 cytokine levels in PBML culture. PBML were isolated from peripheral blood. Cytokine level in PBML supernatants was determined 24 h after incubation with vaccines against influenza by flow cytometry. All data are shown as individual means, M (mean values) ± SD (standard deviation). The significance of differences between mean values at each time point was tested by Mann–Whitney rank sum tests for independent samples (*P* ≤ 0.05).

When exposed to vaccines against influenza (respectively, subunit, immunoadjuvant, and split-), Th-1 cytokine levels were increased compared to the control (PBML supernatant without vaccines): IL-12 level-−10.8, 18.7, and 16.5 times higher compared to control (from 6.0 to 65, 112.4, and 99.4 pg/mL, SD = 12, 21, 7, *p* < 0.001; Figure 1). At the same time, the activity of the immunoadjuvant vaccine was 1.7 times higher relatively to the subunit vaccine (*p* < 0.001) and 1.1 times higher compared to split vaccine (*p* > 0.05).

IFN-γ levels were 15.1, 49.4, and 38.3 times higher compared to the control (from 4.2 to 63.5, 207.5, and 160.9 pg/mL, SD = 9.4, 22, 20, *p* < 0.001). IFN-γ level increase was the least pronounced in response to subunit vaccine compared to other vaccines (*p* < 0.001).

IL-2 levels were12.7, 19.5, and 15 times higher compared to the control (from 7.8 to 99.1, 152.8, and 117.6 pg/mL, SD = 20, 11, 21, *p* < 0.001). There was no statistically significant difference observed between the subunit and split vaccine (*p* > 0.05), in contrast to the immunoadjuvant vaccine, which activated IL-2 production more actively than the subunit (1.5 times higher, *p* < 0.001) and split vaccines (1.2 times higher, *p* < 0.005).

Similar pattern was observed in relation to IL-6 levels which were increased by 6.1, 11.6, and 7.7 times compared to the control (from 19.4 to 119.8, 225.6, and 150.7 pg/mL, SD = 21, 14, 20, *p* < 0.001).

IL-1β levels were 4.8, 12.9, and 7.6 times higher compared to the control (from 31.2 to 150, 403.7, and 240.1 pg/mL, SD = 9.7, 26.9, 15.8, *p* < 0.001). This cytokine level showed maximum increase among all Th-1 cytokines, especially when immunoadjuvant vaccine was used. The concentrations of IL-1β increased by 2.6 and 1.7 times compared with the subunit and split vaccines (*p* < 0.001).

TNF-α levels were 3.8, 15.4, and 12.2 times higher compared to the control (from 16.6 to 63.7, 256, and 204 pg/mL, SD = 7.7, 35, 37.4, *p* < 0.001). There were no significant differences between immunoadjuvant and split vaccines (*p* > 0.05), while these vaccines caused a statistically significant increase in TNF-α levels compared to subunit vaccine (4 and 3.2 times, respectively, *p* < 0.001).

Therefore, all the studied influenza vaccines caused increased levels of all Th-1 cytokines in the PBML culture fluid, which may evidence the cellular immune response activation. At the same time, maximum activity was shown for immunoadjuvant vaccine, lowest activity—for the subunit vaccine, so that, split vaccine was in intermediate position.

**Figure 2:**
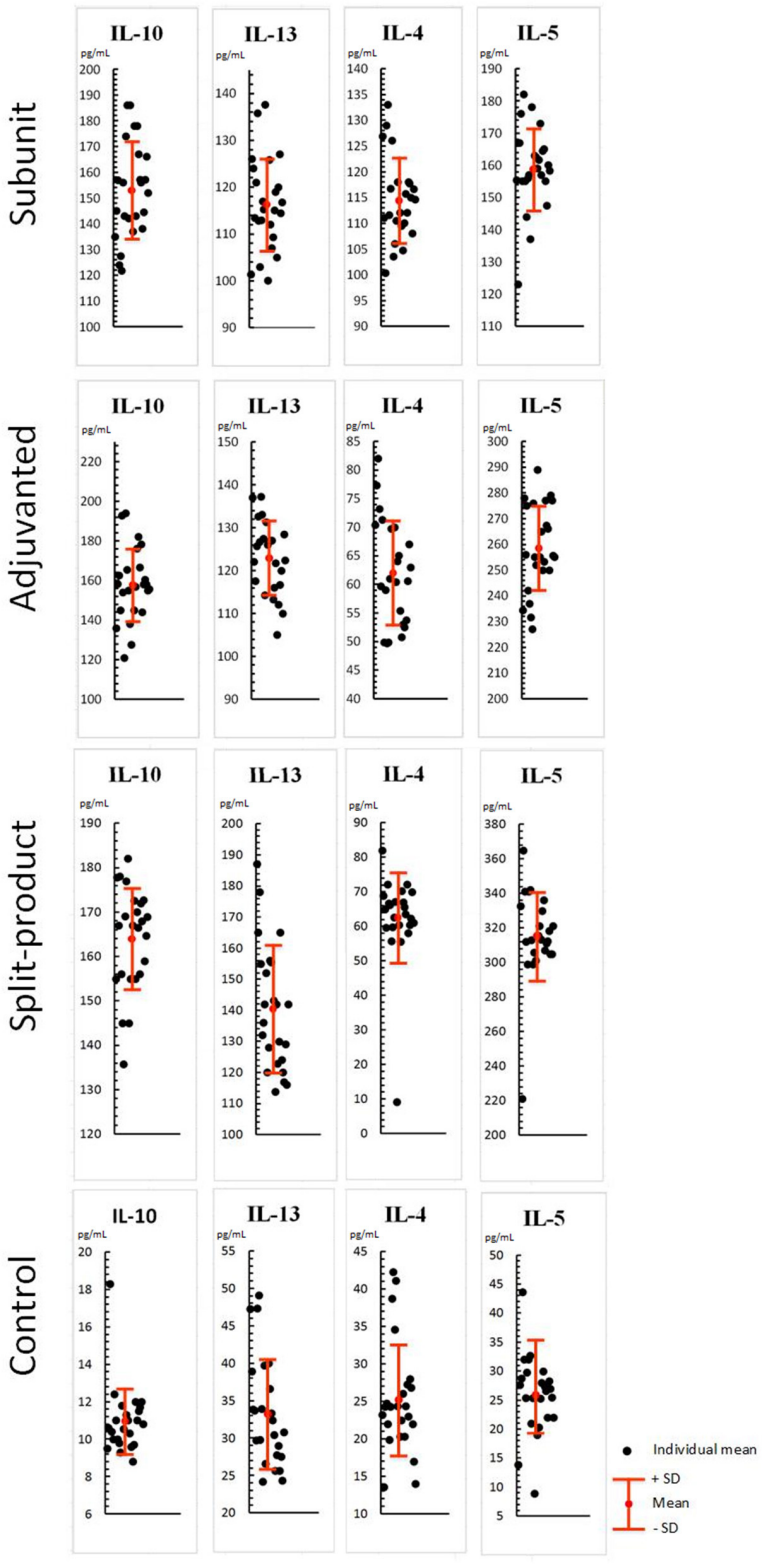
Effect of vaccines against influenza on Th-2 cytokine levels in PBML culture. PBML were isolated from peripheral blood. Cytokine level in PBML supernatants was determined 24 h after incubation with vaccines against influenza by flow cytometry. All data are shown as individual means, M (mean values) ± SD (standard deviation). The significance of differences between mean values at each time point was tested by Mann–Whitney rank sum tests for independent samples (*P* ≤ 0.05).

When exposed to vaccines against influenza (respectively, subunit, immunoadjuvant, and split-), Th-2 cytokine levels were also increased compared to the control (PBML supernatant without vaccines): IL-10 levels were 13.9, 14.3, and 14.9 times higher compared to the control (from 11 to 152.9, 157.7, and 163.9 pg/mL, SD = 18.6, 18.3, 11.6, *p* < 0.001). There were no significant differences between the groups (*p* > 0.05; Figure 2).

IL-13 levels were 3.5, 3.7, and 4.2 times higher compared to the control (from 33.2 to 116.3, 122.9, and 140.4 pg/mL, SD = 9.8, 8.5, 20.1, *p* < 0.001). There were no significant differences observed between subunit, immunoadjuvant, and split vaccines (*p* > 0.05).

IL-4 levels were 4.5, 2.4, and 2.5 times higher compared to the control (from 25.1 to 114.4, 62, and 62.5 pg/mL, SD = 8.1, 9.2, 12.8, *p* < 0.001). The activity of the subunit vaccine was 1.8 times higher than of immunoadjuvant and split vaccines (*p* < 0.001).

IL-5 levels were 6.1, 10, and 12.1 times higher compared to the control (from 25.9 to 158.8, 258.4, and 315.3 pg/mL, SD = 12.7, 16.7, 26, *p* < 0.001). The activity of the split vaccine was significantly higher compared to the subunit (2 times) and immunoadjuvant vaccines (1.2 times), *p* < 0.001.

Therefore, all studied vaccines leaded to increased levels of all Th-2 cytokines in leukocyte supernatants, which indicates humoral immunity activation. Regarding IL-4, it can be concluded that the immunoadjuvant and split vaccine had similar potential, consisting in the increase of this cytokine level by 1.8 times, *p* < 0.001. The subunit vaccine was the most active in regard to IL-4 stimulation. Split vaccine had more pronounced effect on IL-5 production.

**Figure 3:**
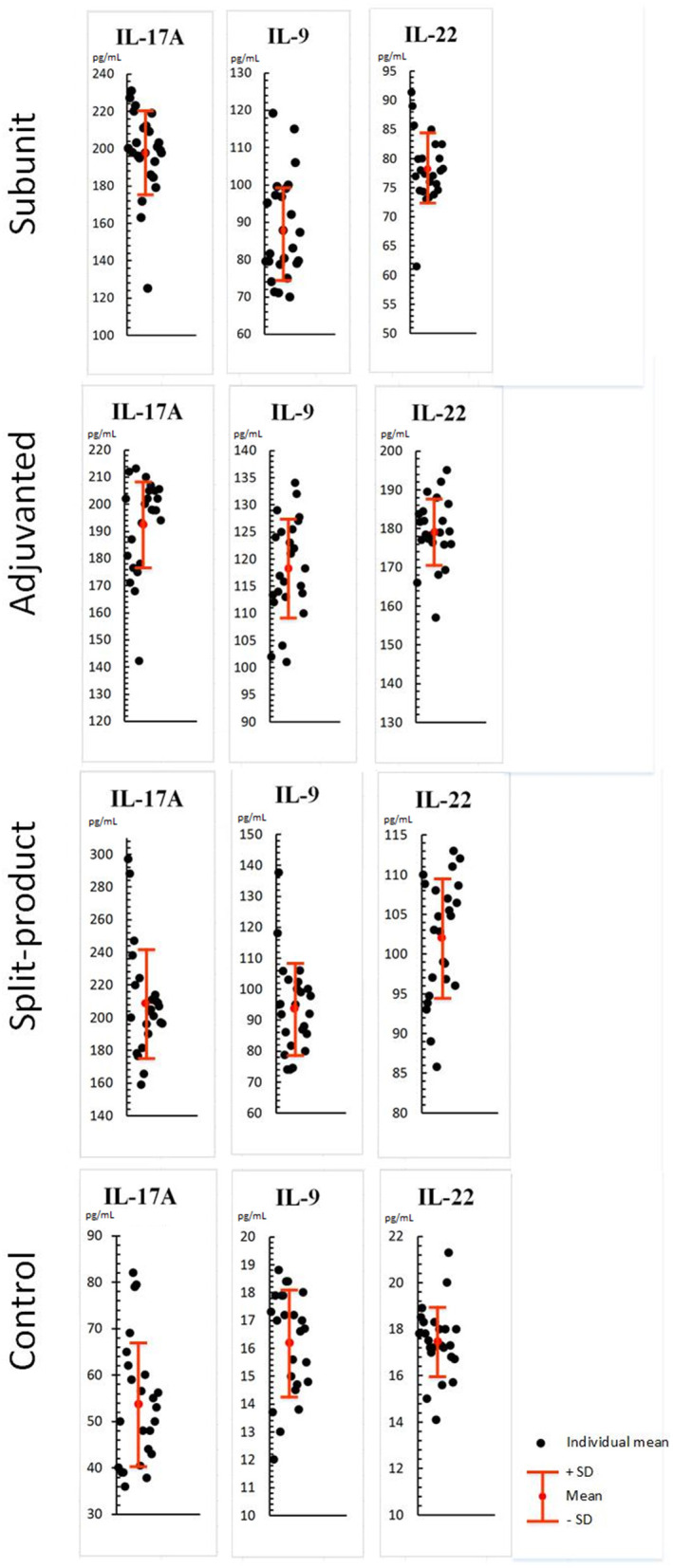
Effect of vaccines against influenza on Th-17/Th-9/Th-22 cytokine levels in PBML culture. PBML were isolated from peripheral blood. Cytokine level in PBML supernatants was determined 24 h after incubation with vaccines against influenza by flow cytometry. All data are shown as individual means, M (mean values) ± SD (standard deviation). The significance of differences between mean values at each time point was tested by Mann–Whitney rank sum tests for independent samples (*P* ≤ 0.05).

When exposed to vaccines against influenza (respectively, subunit, immunoadjuvant, and split-), IL-17A, IL-9, IL-22 levels increased compared to the control (PBML supernatant without vaccines): IL-17A levels were 3.6, 3.5, and 3.8 times higher compared to the control (from 53.8 to 197, 192.6, and 208.9 pg/mL, SD = 22.8, 17.1, 32.8, *p* < 0.001; Figure 3). Difference between experimental groups was not statistically significant (*p* > 0.05).

IL-9 levels were 5.4, 7.3, and 5.7 times higher compared to the control (from 16.2 to 87.9, 118.3, and 93.8 pg/mL, SD = 13.8, 9, 14.7, *p* < 0.001). The activity of the immunoadjuvant vaccine was 1.3 times higher than of the subunit vaccine and 1.2 times higher than of the split vaccine—(*p* < 0.001).

IL-22 levels were 4.5, 10, and 5.8 times higher compared to the control (from 17.4 to 78.2, 179.1, and 102 pg/mL, SD = 6, 8.4, 7.5, *p* < 0.001). The activity of the immunoadjuvant vaccine was 2.2 times higher than of the subunit vaccine and 1.7 times higher than of the split vaccine—(*p* < 0.001). Statistical differences between the subunit and split vaccine groups were identified. The activity of the last one was 1.2 times higher (*p* < 0.05).

All vaccines caused increased levels of IL-17A, IL-9, IL-22 cytokines in the PBML culture fluid. The immunoadjuvant vaccine had a more pronounced potential for IL-9 and IL-22 activation in comparison with subunit and split vaccines.

## Discussion

The development of modern technologies for the production of influenza vaccines using adjuvants can increase the immunogenicity of drug products. This is achieved both by affecting inductor cells and effectors of the immune response. The mechanism of development of the immune response and formation of immunological memory includes a wide variety of interrelated and interdependent processes. An integral component of these processes is cytokines produced during the immune response and creating a certain microenvironment for cells involved in the formation of immune protection. Thus, by evaluating the production of cytokines by immune cells, we can indirectly judge both the activity of these cells, and better understand the necessary cytokine microenvironment that leads to high immunogenicity. And also thanks to such studies, the role of the adjuvant becomes more clear.

The study showed that the immunoadjuvant vaccine containing Azoximer bromide more actively induced the synthesis of Th1 cytokines (IL-12, INF-γ, IL-2, IL-6, IL-1β, TNF-α), while the subunit showed minimal effect on their level in comparison with both the immunoadjuvant and split vaccine. These data correlate with our previous studies. PO-containing adjuvanted vaccine showed the strongest capability of inducing the cellular response, among the three vaccines studied, increases in the number of the innate and acquired immunity effectors: NK cells, NKT cells, B lymphocytes, cells with early activation marker, T lymphocytes with late activation marker, and regulatory T cells (25).

It should be noted that the results of the cytokine profile were evaluated without taking into account the initial antibody titers. However, when evaluating the parameters of the cellular immune response, the following results were obtained: in individuals with initially low antibody titers (titer 1:20–1:40), all types of vaccines demonstrated effectiveness in activating the cellular component of immune system, while in individuals with medium (1:80–1:160) and high antibody titers (1:320 and <) to influenza virus strains, the advantage of the adjuvant vaccine was revealed (25).

IL-12, INF- γ, and IL-2 are better known for their regulatory function, and IL-6, IL-1β, and TNF-α are proinflammatory cytokines, the most potent of which is IL-1β. On the one hand, this may be alarming, on the other hand, analysis of the functions of IL-1β shows its important participation in the formation of a post-vaccination immune response. In particular, it is known that IL-1β is a powerful activator of dendritic cells. IL-1β, participating in increasing CD40L-mediated activation of dendritic cells, increases their ability to control the secretion of IFN-γ in T cells (42) and produce IL-12 (43). Thus, it is possible that the activity of Th2 cells in relation to the production of IL-12, INF-γ is not mediated by IL-1β. In addition, IL-1β directly affects antigen-specific CD8^+^ T cells, enhancing their expansion, survival, migration, and effector function, including granzyme B expression and IFN-γ secretion (44). Thus, IL-1β is equally, and possibly more than, other cytokines, involved in creating an optimal cytokine environment for the formation of a cellular immune response involving DC, CD4^+^, and CD8^+^ cells. Concerns about the development of an inflammatory reaction are not justified, since the use of an adjuvant vaccine as part of routine practice for many years has proven its safety. Moreover, analysis of the results of clinical trials involving an adjuvant vaccine demonstrates a comparable reactogenicity profile and number of systemic reactions compared to a non-adjuvant subunit influenza vaccine.

In terms of activity against IL-4 and IL-5, the adjuvant vaccine took an intermediate place. The subunit vaccine had a stronger effect on IL-4 production, while the split vaccine had a stronger effect on IL-5 production. Producers of IL-4 and IL-5 are also known to be Th2 cells.

It is also known that IL-4 can be synthesized not only by activated T-lymphocytes, but also by basophils and mast cells. The spectrum of biological action covers the suppression of the inflammatory response and increased activation of humoral immune response (45). And the significance of these cytokines increases significantly in the T and B areas of the lymph node. High levels of IL-4 may play a negative role in patients with allergic pathology, and hyperproduction of IL-4 may provoke an anabasis (46–48).

The absence of IL-4 hyperproduction when using an adjuvant vaccine explains the results of clinical studies in which literature analysis shows that when vaccination against influenza with a subunit vaccine with an Azoximer bromide adjuvant in children with allergic diseases, there was a significant decrease in the content of IgM and total IgE in the blood serum after 1 month and there was a tendency to increase endogenous IFN-γ and IL-12 (49). Therefore, the use of the studied immunoadjuvant vaccine was not accompanied by an increase in sensitization in individuals with allergic pathologies.

These results of the study have shown a more significant effect of the immunoadjuvant vaccine on IL-9 and IL-22 levels in comparison with subunit and split vaccines. The cytokine IL-9 was identified and its main characteristics were described more than three decades ago. Earlier, IL-9 was considered to be Th2-cytokine, as it promotes the development of allergic inflammation and is associated with various Th2-type responses (50). Later studies have shown the multifunctional activity of this cytokine. IL-9 turned out to be a key molecule, that affects Th17 cell differentiation and T-reg functions. IL-9 enhances the suppressor function of Foxp3^+^CD4^+^T-regs *in vitro*, and absence of IL-9 signaling reduces the inhibitory activity of T-regs *in vivo*, which leads to increased number of effector cells. IL-9 is secreted by a recently discovered CD4^+^T helper subpopulation—Th9-cells that differ from Th1, Th2, and Th17 (51). It should be noted that immunoadjuvant-containing vaccine was the only one that increased T-regs number (25). It can be assumed that a more pronounced stimulation of this cytokine in combination with an increase in the number of T-reg happening under the influence of an immunoadjuvant vaccine, has a regulating effect on the immune response, preventing immune system hyperactivation.

IL-22 exerts functions, similar to IL-17; both cytokines contribute to the control of extracellular bacterial infections (52). The main biological role of IL-22 includes the enhancement of innate immunity, protection from damage, as well as increase regeneration (51).

Thus, increased IL-9 and IL-22 levels may indicate increased cellular regulatory functions in the immune response and activation of the innate mechanisms of the immune system in response to influenza vaccines, in particular, containing immunoadjuvants.

## Conclusion

Studied vaccines against influenza were associated with increased levels of Th1/Th2/Th17/Th9/Th22 cytokines in the PBML culture fluid, which indicates both humoral and cellular immunity activation. Immunoadjuvant vaccine is superior in terms of inducing cellular immune effectors to a greater extent compared to subunit and split vaccines.

## Data Availability Statement

The datasets generated for this study are available on request to the corresponding author.

## Ethics Statement

The studies involving human participants were reviewed and approved by Ethics committee in I.I. Mechnikov Research Institute of Vaccines and Sera, Moscow, Russia. The patients/participants provided their written informed consent to participate in this study.

## Author's Note

The vaccines used comply with biosafety and biosecurity standards, which is confirmed by the marketing authorizations of the drug product for medical use.

## Author Contributions

NA, EK, and MK: concept and design of the study. EK and AK: collection and processing of material. NA: statistical processing. All authors: contributed to the article and approved the submitted version.

## Conflict of Interest

The authors declare that the research was conducted in the absence of any commercial or financial relationships that could be construed as a potential conflict of interest.

## Abbreviations

PBML: peripheral blood mononuclear leukocytes
DCs: dendritic cells
PO, Polyoxidonium: Azoxymer bromide
T-reg: T-regulatory cells.
